# Automated Microfluidics for Efficient Characterization of Cyclohexanol Electrooxidation for Sustainable Chemical Production

**DOI:** 10.1021/jacsau.4c01207

**Published:** 2025-03-04

**Authors:** Xiao Liang, Mengzheng Ouyang, Nigel P. Brandon, Jin Xuan, Huizhi Wang

**Affiliations:** † Department of Mechanical Engineering, Imperial College London, London SW7 2AZ, U.K.; ‡ Department of Earth Science and Engineering, Imperial College London, London SW7 2AZ, U.K.; § School of Chemistry and Chemical Engineering, Faculty of Engineering and Physical Sciences, University of Surrey, Guildford GU2 7XH, U.K.

**Keywords:** microfluidics, electrocatalysis, characterization, automation, cyclohexanol oxidation, power-to-X

## Abstract

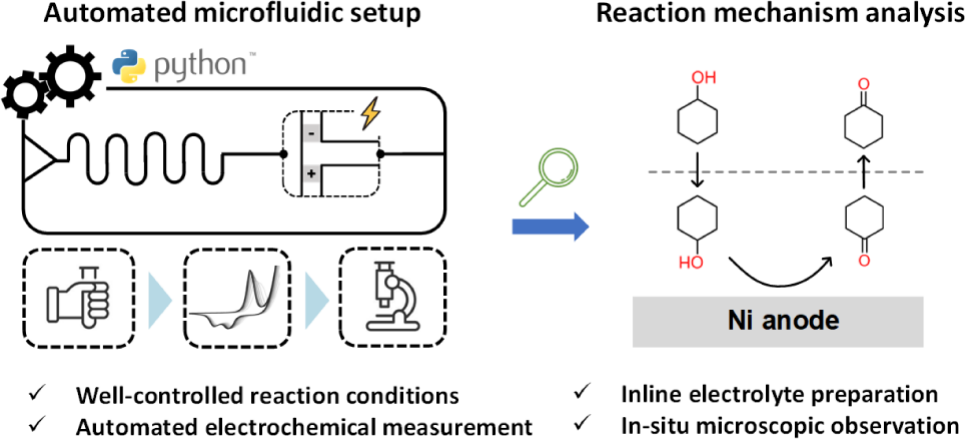

The electrochemical conversion of biomass-derived compounds into value-added chemicals using renewable electricity has attracted attention as a promising pathway for sustainable chemical production, with the electrooxidation of cyclohexanol being a typical example. However, optimizing and upscaling these processes have been hindered due to a limited understanding of the underlying mechanisms and limiting factors. To address this, there is a critical need for experimental tools that enable more efficient and reproducible measurements of these complex processes. In this work, we develop an automated microfluidic platform and use it to conduct controlled and efficient measurements of cyclohexanol electrooxidation on nickel electrodes under various electrolyte compositions and flow rates. The platform features microchannel networks integrated with multiple analytical instruments such as pumps, an electrochemical workstation, and a digital microscope to perform laboratory functions including electrolyte preparation, reaction control, microscopy, and electrochemical characterization, all streamlined through automation. Cyclohexanol electrooxidation on nickel is found to follow Fleischmann’s mechanism, with an irreversible heterogeneous reaction as the rate-determining step. The effects of ionic and nonionic surfactant additives are screened, both demonstrating the ability to enhance current densities through different mechanisms. The developed platform is readily transferable for measuring other power-to-chemical processes and is believed to be a powerful tool for accelerating the understanding and development of sustainable electrosynthesis.

## 1 Introduction

Power-to-chemicals, which converts renewable electricity to chemical products, has been attracting significant attention as a promising solution for decarbonizing the hard-to-abate chemical sectors while providing long-term storage for surplus renewable supply.^1−4^ A notable example of “power-to-chemicals” is the electrooxidation of cyclohexanol to cyclohexanone, a key precursor in the production of nylon polymers, pharmaceuticals, and fine chemicals.^5−8^ Due to thermodynamic constraints, conventional catalytic oxidation of cyclohexanol often requires harsh oxidants and energy-intensive conditions, while also struggling with selectivity, leading to high waste and carbon emissions.^8−10^ In contrast, the electrooxidation of cyclohexanol has been demonstrated to occur under ambient conditions and provide better control of oxidation and thus improved selectivity compared to conventional methods.^5,6,11^ When implemented, the electrooxidation of cyclohexanol can pair with a hydrogen-evolving cathode, replacing the sluggish oxygen-evolving anode of water electrolysis to enable more energy-efficient coproduction of hydrogen. Since cyclohexanol can be derived from lignin biomass,^12^ its electrooxidation to cyclohexanone and subsequent conversion to caprolactam and adipic acid can potentially present a sustainable path for chemical production.

Despite the advantages of electrooxidation of cyclohexanol, the underlying reaction mechanisms and limiting factors are not yet fully understood,^13^ with inconsistencies in the literature. These inconsistencies have hindered optimization and created uncertainties about the scalability and industrial applicability of this process, largely attributed to the different cell types and designs used in the different studies. Johann et al.^14^ used a double-walled cylindrical cell to study the electrooxidation of cyclohexanol on a Ni-based electrode in a NaOH electrolyte, achieving a current density of 6 mA/cm^2^ and a cyclohexanone yield of 65%. With the same electrode material and electrolyte, Chaenko et al.^15^ reported a current density approximately 4 times higher using a smaller-volume undivided cell; however, this was at the cost of the ring-opening oxidation of the generated cyclohexanone into adipic acid. Wolfgang et al.^5^ reported a cyclohexanone yield of up to 90% using a batch cell with high-speed stirring. The inconsistencies are also present in other “power-to-chemicals” studies due to the variations in the testing cells used.^16−19^ These variations could lead to different transport and reaction conditions,^20−22^ which may be misattributed to electrode properties and processes, making the results noncomparable between laboratories. Therefore, reactor designs that ensure consistent and reproducible conditions are critical for obtaining meaningful and comparable results. Moreover, the understanding of the electrochemical process is further complicated by various factors such as electrode materials, electrolytes, and additives. The high dimensionality of the problem makes measurements time-consuming and labor-intensive.

In this study, we develop a novel methodology for the controlled and efficient study of the electrooxidation of cyclohexanol using an automated microfluidic platform. By manipulating fluids at the microscale, microfluidics offers precise control over transport processes and reaction conditions.^23^ Its laminar flow characteristics can naturally separate the anolyte and catholyte streams, facilitating independent analyses of reactions at the anode and cathode, as well as a detailed understanding of performance-limiting factors. As a key enabler of lab-on-a-chip systems, microfluidics also allows for the integration of various laboratory functions onto a single chip. Our approach involves designing microchannel networks to perform essential functions for studying cyclohexanol electrooxidation, including electrolyte preparation, reaction control, and characterization, integrating these into a single platform, and streamlining their operations through automation. All components are controlled and synchronized by a Python program. Through combined electrochemical and microscopy analyses, we validate the mechanism of cyclohexanol oxidation on nickel (Ni)-based electrodes, identify the rate-determining step, and screen the effects of different surfactant additives. The platform can be readily transferred for the study of other power-to-chemicals processes.

## 2 Experimental Platform and Methods

### 2.1 Design of Microfluidic Devices

**Figure 1:**
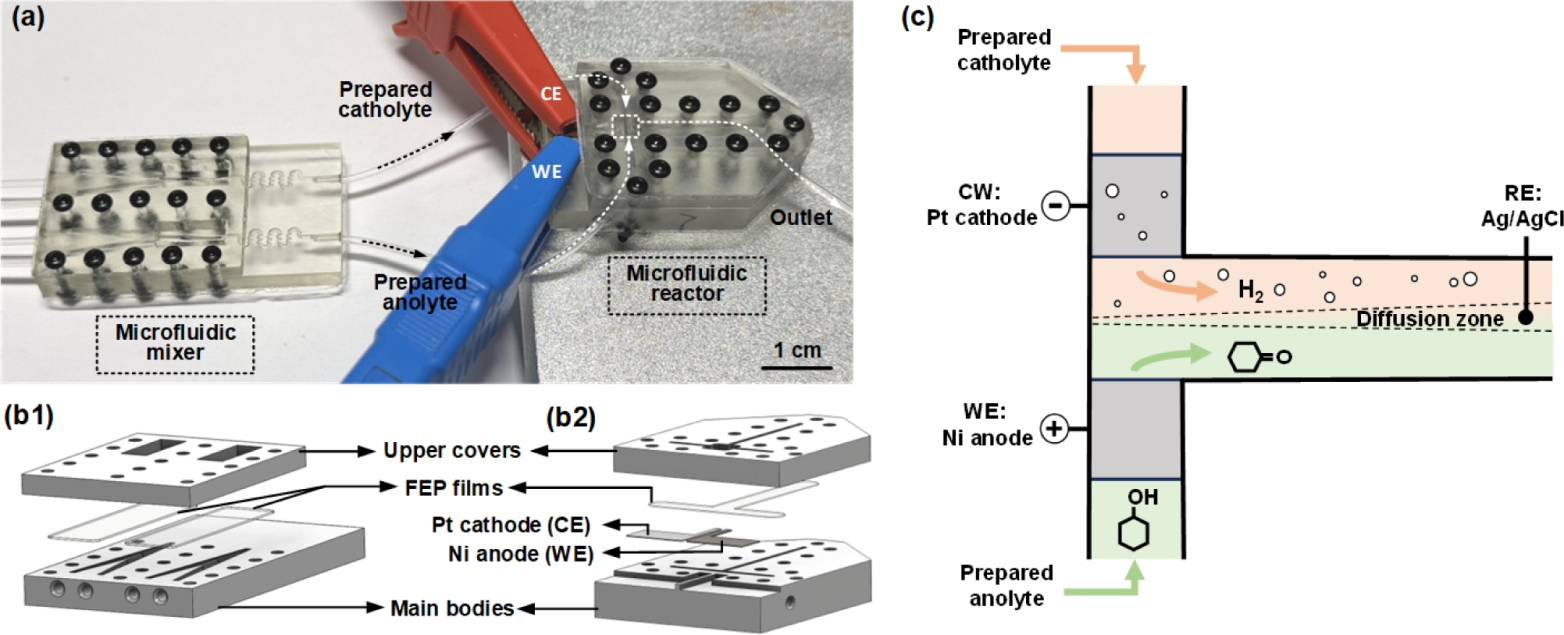
Electrochemical microfluidic devices for investigating cyclohexanol electrooxidation. (a) A photo of a 3D-printed micromixer integrated with a microfluidic electrochemical reactor. Flow paths inside device are illustrated by dashed lines with arrows; (b) structures of the (b1) micromixer and (b2) microfluidic electrochemical reactor; and (c) schematic showing the working principle of the T-shape counter flow microfluidic electrochemical reactor.

The microfluidic platform consisted of a micromixer and an electrochemical reactor designed for inline preparation of electrolytes and subsequent electrochemical measurements, with a photo shown in Figure 1a. The mixer was fabricated by a microfluidic 3D printer (H50, CADworks3D) for preparing electrolytes with varying compositions by inline mixing the concentrated electrolytes and the dilution solutions. It consisted of a 3D-printed main body, as shown in Figure 1b1, with specially designed fluidic microchannels for enhancing mixing, whose image is shown in Figure S1, and holes for tube and screw assembly. Two 0.3 mm thick fluorinated ethylene propylene (FEP) transparent films were placed above the microchannels, sandwiched between the main body and a 3D-printed upper cover to prevent leaking. The electrochemical reactor employed a T-shape counterflow design, as illustrated in Figure 1b2,c. Ni was used as the working electrode for cyclohexanol oxidation, while platinum (Pt) was used as the counter electrode, where the hydrogen evolution reaction occurred to complete the circuit. Both electrodes had active areas of 1 × 0.8 mm^2^, and they were arranged in a coplanar configuration with an observation window positioned above to facilitate in-operando microscopy analyses. An Ag/AgCl (saturated KCl) electrode was positioned downstream of the outlet tube as a reference electrode. As shown in Figure 1b2, the reactor contained fluidic channels (0.8 mm width × 0.8 mm depth) for electrolytes and devised pockets for electrode integration. The junctions of electrodes and tubes with the main body were sealed by applying a small amount of 3D-printing resin and polymerizing it under UV light for 10 s. The total cost of these two microfluidic devices was less than 15 pounds, much cheaper than the commercial H-cell.

Sodium hydroxide (NaOH, 98%, from Sigma-Aldrich) solution containing dissolved cyclohexanol (99%, from Sigma-Aldrich) served as the anolyte. Three surfactants were used as additives in the anolyte to investigate their effects on cyclohexanol oxidation, including cetyltrimethylammonium bromide (CTAB, 98%, from Sigma-Aldrich), sodium dodecyl sulfate (SDS, 99%, from Sigma-Aldrich), and poly(ethylene glycol) *p*-(1,1,3,3-tetramethylbutyl)-phenyl ether (Triton X-100, from Sigma-Aldrich). A NaOH solution without cyclohexanol was used as the catholyte. The anolyte and the catholyte, with their respective compositions tuned in line, were supplied through the micromixer to the anode and cathode in the electrochemical reactor. Under laminar flow conditions, the two electrolyte streams flowed side by side in the electrochemical reactor without bulk mixing, effectively preventing the undesired diffusion of reactants and products to the opposite electrode, thereby ensuring accurate measurements.

### 2.2 Automated Microfluidic Platform

**Figure 2:**
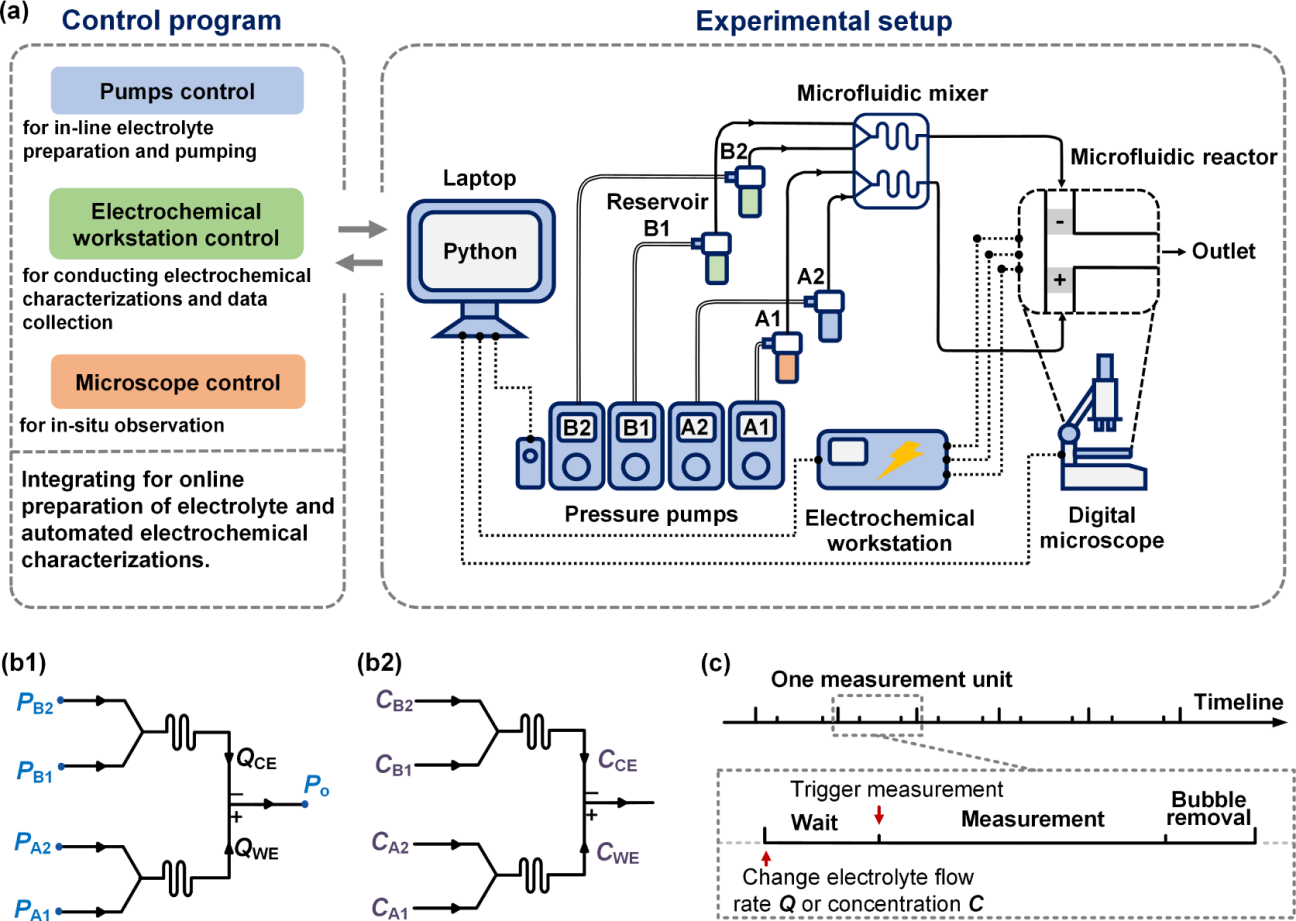
Microfluidic experimental platform and automation design. (a) Microfluidic setup and control program. Solid lines with arrows represent the fluid flow and direction, double solid lines represent the compressed gas for pumping fluids, and shot-dashed lines with dots represent the communication between setups for control and data collection. (b) Principles for inline adjusting the electrolyte (b1) flow rate and (b2) compositions. (c) Procedures of automated multimeasurements.

The entire experimental platform is shown in Figure 2a. Pressure pumps (Flow EZ, Fluigent) were used to control the flow rates. The continuous operation of the pumps combined with large reservoirs allows for pulseless flow for long-term measurements without the need to refill the reagents. Two pumps, A1 and A2, were used to pump the solutions stored in reservoirs (250 mL bottles with airtight pressurized caps) A1 and A2 into the micromixer. Two solutions were mixed in the micromixer to prepare the anolyte and flowed into the electrochemical reactor. Two other pumps, B1 and B2, were used to pump the catholyte stored in reservoirs B1 and B2. All four pumps were connected to a laptop by a controller module (LineUp LINK, Fluigent) using USB communication, and the pressure source was supplied stably by a compressed air generator (FLPG Plus, Fluigent), which is not shown in the schematic. An electrochemical workstation (CHI 660E, CH Instruments) was used for electrochemical measurements. A digital microscope (DM022B, Topnisus) was used for in-operando optical observation.

As detailed in Figure 2b, the inline preparation of electrolytes with varying compositions was automated by adjusting the outlet pressures of the four pressure pumps. The electrolyte concentrations *C*_WE_ and *C*_CE_ can be adjusted from *C*_A1_ to *C*_A2_ and from *C*_B1_ to *C*_B2_ by tuning *P*_A1_ and *P*_A2_ and *P*_B1_ and *P*_B2_, respectively, as shown in Figure 2b1,b2. *P*_A1_ + *P*_A2_, and *P*_B1_ + *P*_B2_ were kept constant to ensure that *Q*_WE_ and *Q*_CE_ remained unchanged. Specifically, for the anolyte, where the composition effect is of interest, liquid A2 served as the dilution solution with *C*_A2_ equal to zero. Thus, *C*_WE_ was calculated as ρ*C*_A1_, where ρ was the dilution factor. ρ was calibrated based on *P*_A1_, as detailed in Supporting Information.

Automated experimentation was achieved by connecting the four pumps, electrochemical workstation, and microscope to a laptop controlled by a Python program. The Python code (provided in Supporting Information) coordinates different platform functions to achieve automated multimeasurements. For each measurement, the procedure included three stages, as shown in Figure 2c. At the beginning of the first stage, *Wait*, outlet pressures were adjusted to prepare the electrolyte with the desired concentration and flow rate, subsequently waiting for a period to allow flow and mixing to stabilize. The waiting time was set to at least two times longer than the resistance time of fluid to pass through the tube. In the second stage, *Measurement*, the electrochemical workstation was activated to start the measurements. After the completion of each measurement, hydrogen bubbles generated at the counter electrode would trap on the channel walls and electrodes. Therefore, in the third stage, four outlet pressures were increased to remove these bubbles at a high total flow rate of 13.2 mL/min.

### 2.3 Electrochemical Measurements and Electrode Pretreatment

The cyclic voltammogram (CV) measurements were performed over a potential range from 0.20 to 0.54 V vs Ag/AgCl (saturated KCl), with scan rates of 5 mV/s–200 mV/s. The linear sweep voltammogram (LSV) measurements were performed over the same potential with a slower scan rate of 2 mV/s. Before measurements, the fresh Ni electrode was pretreated in situ using a 0.5 mol/L NaOH solution to generate a hydroxide layer on the surface. The method was to run CVs from 0.4 to 1.7 V vs Ag/AgCl (saturated KCl) with a scan rate of 100 mV/s for 200 cycles. This electrochemical treatment can result in the formation of a stable β-Ni(OH)_2_ layer on the nickel surface,^13^ which worked as the Ni^2+^/Ni^3+^ redox couple for cyclohexanol oxidation. All experiments were performed at ambient temperature (25 ± 1 °C).

## 3 Results and Discussion

### 3.1 Setup Calibration

**Figure 3:**
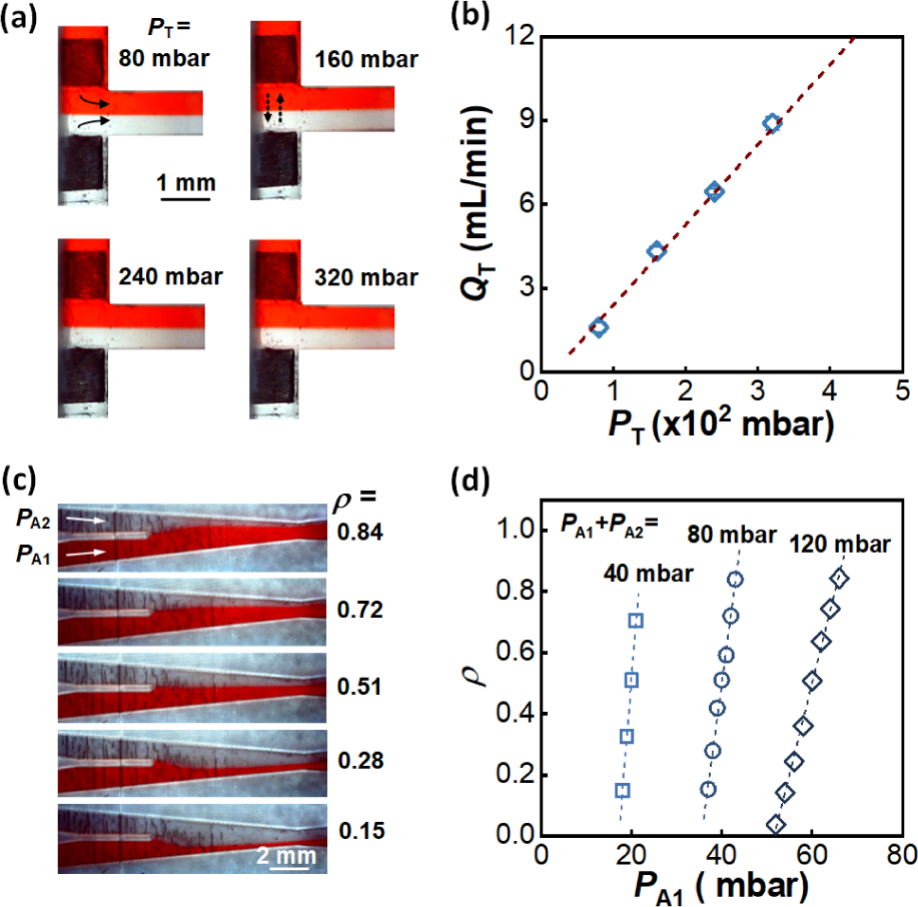
Flow rate calibration and dilution factor calibration. (a) Flow configurations with different total outlet pressure *P*_T_. *P*_A1_ + *P*_A2_ is equal to *P*_B1_ + *P*_B2_ in the study, so *Q*_WE_ is equal to *Q*_CE_, and the diffusion zone always stays at the centerline of the main channel. Solid lines with arrows represent the flows of two fluids in the junction region. Short-dash lines with arrows represent the exchange of species in two fluids. 0.1 wt % red dye was added to differentiate between two fluids. (b) The relationship between *P*_T_ and total flow rate *Q*_T_, *Q*_T_ = 2*Q*_WE_ = 2*Q*_WC_. (c) The images of two fluids intersecting in the micromixer with different dilution factors. (d) Relationships of dilution factor ρ and outlet pressure *P*_A1_ at different sums of *P*_A1_ and *P*_A2_.

The species exchange between the anolyte and catholyte was first visually investigated by using a red dye solution, as shown in Figure 3a. Examinations were conducted with the total outlet pressure *P*_T_ ranging from 80 to 320 mbar, resulting in the total flow rates *Q*_T_ from 1.8 to 8.8 mL/min. A linear relationship between *P*_T_ and *Q*_T_ was observed, as shown in Figure 3b. In this range, the formation of parallel flow was stable, and no red dye exchange was observed between the two streams in the junction region. The Peclet number (Pe) was used to characterize the relative importance of convection to diffusion,
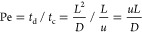
1where *t*_d_ is the diffusion time scale and *t*_p_ is the convection time scale of fluids to pass through the junction region; *L* is the characteristic length, set as the length of the junction region, measured as 0.8 mm; and *u* is the average velocity of fluid in the branch channel, ranging from 1.6 × 10^–2^ m/s to 1.2 × 10^–1^ m/s. For common molecules in aqueous solutions, *D* ranges from 1 × 10^–10^ m^2^/s to 10 × 10^–9^ m^2^/s. Pe was calculated to fall within the range from 1.2 × 10^3^ to 9.2 × 10^5^, much larger than 1. This suggested that the molecule diffusion between two fluids was much slower compared to the convection in bulk fluid flow. Since the two electrodes were located on either side of the junction region, crossover that could affect the electrochemical measurements can be ignored. Given the partial ionization of cyclohexanol in alkaline aqueous solution, the migration process was examined by using the convection-migration (CM) number. This number characterizes the relative strength of convection to migration in an electric field,
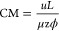
2where μ is the mobility of ions, estimated to from 1.0 × 10^–8^ m^2^/V·s to 1.0 × 10^–7^ m^2^/V·s for common ions, *z* is the charge number of ions, and ϕ is the applied potential estimated to be 2 V. The CM number was calculated to fall within the range from 1.3 × 10^2^ to 9.2 × 10^3^, large enough to minimize the effect of ion migration from one electrode to another.

When the total outlet pressure was over 320 mbar, the Reynolds number (Re), which measured the relative importance of inertial forces compared to viscous forces, was higher than 200,
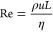
3where ρ and η are the density and the dynamic viscosity of the fluid. Such a large Re number indicated that the inertial forces became dominant, tending to keep the two fluids flowing in the opposite directions along the branch channel and exacerbate the undesired convective mixing in the junction region,^24,25^ which was consistent with the experimental observations. Therefore, the maximum safe operational total outlet pressure was set to 320 mbar, and the total flow rate was 8.8 mL/min.

The dilution factor in our microfluidic platform design was further examined. Figure 3c shows the images of two fluids intersecting in the micromixer. Liquid A1 with 0.1 wt % red dye was used to simulate the concentrated electrolyte solution, and liquid A2 served as the dilution solution. The diluted solution was collected at the final outlet to calculate the dilution factor. Two fluids were pumped into channels by outlet pressures *P*_A1_ and *P*_A2_. By adjustment of *P*_A1_ and *P*_A2_ with keeping *P*_A1_ + *P*_A2_ unchanged, the ratios of flow rates of the two fluids were changed, leading to different dilution factors ρ. The relationships between ρ and *P*_A1_ were linear, which were also related to the sum of *P*_A1_ and *P*_A2_, as shown in Figure 3d. Results demonstrated that the concentration control range was from 0.1 to 0.9 times the concentration of the concentrated solution, following different linear relationships with different outlet pressures. More detailed analysis can be found in Supporting Information.

### 3.2 Electrochemical Behaviors of the Ni Electrode in the NaOH Solution

**Figure 4:**
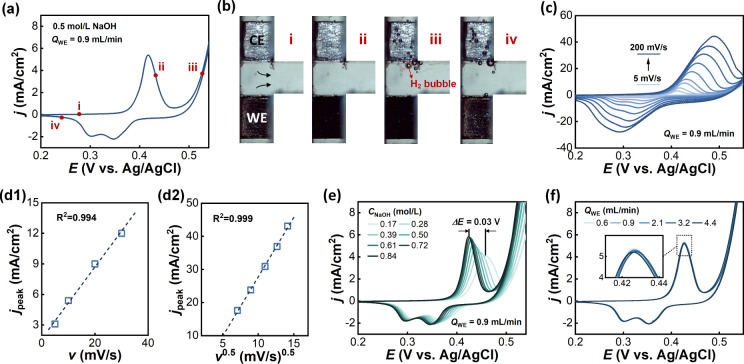
Cyclic voltammograms on the nickel electrode in 0.5 mol/L NaOH solution and the corresponding images at four typical moments. (a) Typical cyclic voltammogram scanning from 0.20 to 0.54 V vs Ag/AgCl (sat. KCl) at 10 mV/s. The flow rate *Q*_WE_ is 0.9 mL/min. (b) The captured images at four typical moments. One hydrogen bubble is highlighted by the red line. (c) The effect of scan rate. (d) The relationships between (d1) peak current densities and scan rates and (d2) peak current densities and the square root of scan rates. (e) The effect of NaOH concentration. (f) The effect of flow rate *Q*_WE_.

Before studying the cyclohexanol oxidation reaction, the electrochemical behaviors of the Ni electrode in 0.5 M NaOH solution without cyclohexanol were first examined to test the platform’s ability for electrochemical characterization. The CV curve is shown in Figure 4a, and the optical images recorded at four typical stages are shown in Figure 4b. The applied potential was limited below 0.54 V vs Ag/AgCl (sat. KCl), i.e., 1.54 V vs RHE, to avoid the oxygen evolution reaction, which was observed to start at about 1.6 V vs RHE. At the low applied potential, no reaction was visible, and the current was close to zero. With increasing potential, an oxidation peak was observed at approximately 0.42 V vs Ag/AgCl (sat. KCl). This peak represented the oxidation of β-Ni(OH)_2_ to β-NiOOH, which was partially converted to another crystallographic phase, γ-NiOOH.^26^ In the visual investigation, the electrode surface gradually turned to a nonglossy deep black due to the accumulation of nickel oxyhydroxide on the surface,^27^ as shown at moment ii. At more positive potentials, hydrogen bubbles started to form at the Pt electrode, and some small bubbles detached from the electrode surface due to the shear forces of the catholyte and flowed away with the electrolyte, as shown at moment iii. During the backward scan, two reduction peaks were observed at approximately 0.35 and 0.30 V vs Ag/AgCl (sat. KCl), indicating the formation of two different phases of nickel oxyhydroxide, probably due to the reduction of β-NiOOH into β-Ni(OH)_2_ and the reduction of γ-NiOOH into the unstable α-Ni(OH)_2_ which was then further transformed to β-Ni(OH)_2_, according to previous studies.^28,29^ At the end of the backward scan, the nickel surface was recovered with the deep black color fading away, as shown in moment iv.

Figure 4c shows CV profiles at various potential scan rates from 5 to 200 mV/s. Increasing the scan rate led to higher current densities in both forward and backward scans due to a decrease in the thickness of the diffusion layer. In the range of low scan rates from 5 to 30 mV/s, the peak current densities *j*_peak_ increased linearly with the scan rates, as shown in Figure 4d1. On increasing the scan rate to 50–200 mV/s, peak current densities increased linearly with the square root of the scan rates, as shown in Figure 4d2. This difference between low and high scan rates was also reported in the literature,^30^ suggesting a transition of the rate-determining step to the dominance of a diffusion-controlled process. CV measurements with different NaOH concentrations and flow rates were then conducted. The anodic peak of β-Ni(OH)_2_ was shifted to the lower potential with increasing NaOH concentration, as shown in Figure 4e. The change in pH from 0.17 to 0.84 mol/L NaOH was about 0.58; thus, the peak potential change Δ*E* can be calculated as Δ*E* = ΔpH = 0.03 V, which agreed well with the experimental value. Increasing the flow rate from 0.6 to 4.4 mL/min had little effect on CV curves, as shown in Figure 4f, indicating that mass transport in the bulk phase was not the rate-determining step for the Ni(OH)_2_/NiOOH redox reaction.

This discussion verified the platform’s ability to perform electrochemical measurements under various controlled conditions, including the electrolyte concentrations and flow conditions. The synchronously captured images can reveal the changes on the Ni electrode surface and the generation of bubbles during the CV measurement, providing a more intuitive understanding of the electrochemical reaction process. All of these data and images in Figure 4 were collected using the automated platform with no human intervention. The automation eliminated the need for time-consuming and error-prone manual tasks, such as electrolyte preparation and replacement, device reassembly, and parameter adjustments during the experiment. As detailed in Figure S3a, this resulted in an approximately 48% reduction in labor time in the experiment compared to the manual operation using the H-cell, highlighting its advantage of time efficiency.

### 3.3 Understanding the Mechanism of Cyclohexanol Oxidation on the Ni Electrode

To further examine the platform’s ability to perform electrochemical characterizations of power-to-chemical reactions and aid in the mechanistic understanding of reaction processes, we used the platform to study the electrochemical oxidation of cyclohexanol on the Ni electrode. The oxidation of organic compounds on nickel electrodes in alkaline solution is commonly described as an indirect heterogeneous process in the literature,^13,31,32^ where nickel oxyhydroxide serves as the electrocatalyst to oxidize the organic compounds irreversibly and is continuously quasi-reversibly reformed on the electrode surface. According to the classical Fleischmann’s mechanism,^33^ the reaction mechanism can be expressed as
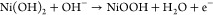
R1
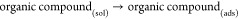
R2
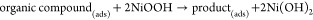
R3
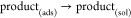
R4

**Figure 5:**
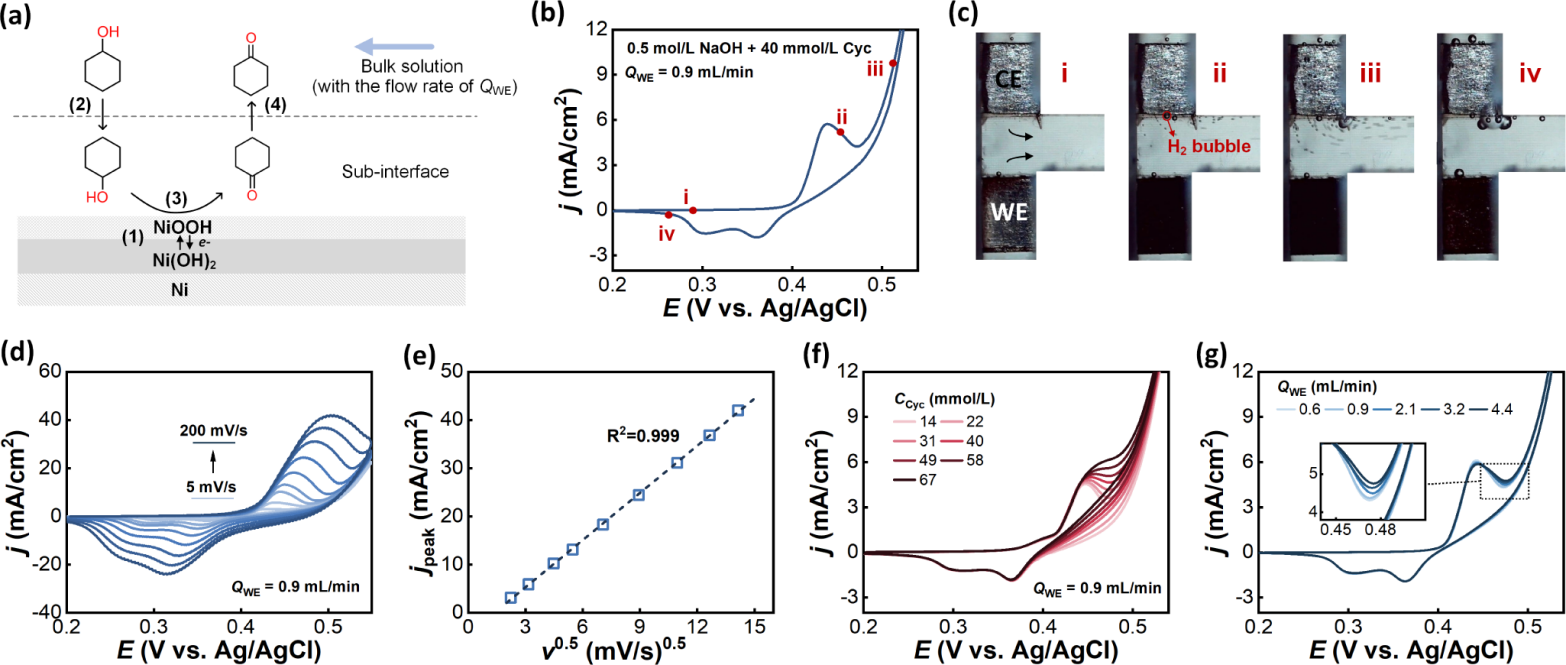
Electrocatalytic oxidation of cyclohexanol on the nickel electrode in NaOH solution. (a) Schematics of electrocatalytic oxidation of cyclohexanol on the nickel electrode in a flowing electrolyte. (b) Typical cyclic voltammogram in 0.5 mol/L NaOH solution containing 40 mmol/L cyclohexanol scanning from 0.20 to 0.54 V vs Ag/AgCl (sat. KCl) at 10 mV/s. The flow rate of the working electrolyte is 0.9 mL/min. (c) The captured images at four typical moments. (d) The effect of scan rate. (e) The relationship between peak current densities and scan rates. (f) The effect of cyclohexanol concentration. (g) The effect of flow rate.

For the oxidation of cyclohexanol, the entire process can be divided into four steps, as shown in Figure 5a, including (1) charge transfer reaction, where nickel hydroxide was electrochemically oxidized into nickel oxyhydroxide; (2) transfer and adsorption of the organic compound, where cyclohexanol molecules transferred from the flowing bulk solution to the subinterface and adsorbed on the nickel oxyhydroxide surface; (3) heterogeneous chemical reaction, where absorbed cyclohexanol was oxidized into cyclohexanone on the nickel oxyhydroxide sites and nickel oxyhydroxide was reduced to nickel hydroxide; and (4) desorption and transfer of the product, where cyclohexanone molecules desorbed from the electrode surface and transferred to the bulk solution.

Figure 5b shows the typical CV profile at a nickel electrode in NaOH solution containing 40 mM cyclohexanol. In the forward scan, the addition of cyclohexanol shifted the β-Ni(OH)_2_ oxidation peak to a more positive potential at 0.44 V vs Ag/AgCl (sat. KCl). The oxidation current densities from 0.44 to 0.54 V vs Ag/AgCl (sat. KCl) increased up to 7 times higher than that without cyclohexanol, indicating a strong electrocatalytic activity of the nickel electrode toward cyclohexanol oxidation. Consequently, more hydrogen bubbles were generated, as shown at moments ii and iii in Figure 5c, demonstrating that the presence of cyclohexanol can facilitate hydrogen evolution. In the backward scan, significant oxidation currents were observed at potentials higher than 0.41 V vs Ag/AgCl (sat. KCl). Two reduction peaks of nickel oxyhydroxide were observed at the same potentials as in the absence of cyclohexanol, but the peak current densities decreased by about 25% on average, probably because some nickel oxyhydroxide was consumed in advance to oxidize cyclohexanol. The observed increase in oxidation currents and the decrease in reduction currents were consistent with Fleischmann’s study,^33^ suggesting that the process was most probably a quasi-reversible charge-transfer reaction followed by an irreversible heterogeneous chemical reaction. It is also interesting to note that the electrode surface was observed to be unable to fully recover at the end of the scan, as shown at moment iv in Figure 5c. This indicated some nickel oxyhydroxide was left on the surface, which was probably mainly due to the slow rate of cyclohexanol oxidation.

To further identify the rate-determining step, CVs at different potential scan rates, cyclohexanol concentrations, and electrolyte flow rates were examined. Figure 5d shows the CVs in 0.5 mol/L NaOH solution containing 40 mM cyclohexanol at different scan rates from 5 to 200 mV/s. The current densities in both forward and backward scans increased rapidly with the increasing potential scan rates. Also, the oxidation peak potential shifted to more positive values, and the reduction peak potentials moved in a negative direction. The CV profiles regained some quasi-reversibility at high scan rates, indicating that the Ni(OH)_2_/NiOOH redox step outperformed the cyclohexanol oxidation step. This was mainly because the cyclohexanol oxidation step was slower than the redox step, resulting in the disappearance of the feature for cyclohexanol oxidation in the CV curves at high scan rates. The linear relationship between the peak oxidation current densities and the square root of scan rates was found across the entire range of scan rates, as shown in Figure 5e. The change in the relationship at low scan rates, compared to that without cyclohexanol, was due to the adsorbed cyclohexanol facilitating the Ni(OH)_2_/NiOOH redox reaction, thereby making the diffusion process more dominant. Figure 5f shows CV profiles with different bulk concentrations of cyclohexanol at a 10 mV/s scan rate. In the forward scan, at potentials more positive than the peak potential of Ni(OH)_2_ oxidation, the current densities increased with increasing cyclohexanol concentration, consistent with Fleischmann’s mechanism. With a higher cyclohexanol concentration, more cyclohexanol molecules were transferred to and adsorbed on the nickel surface, producing more Ni(OH)_2_ sites by reaction (R3) to be oxidized. This was favorable for reaction (R1) to occur at a higher rate, i.e., with a higher current density. On the other hand, a high surface coverage of cyclohexanol may hinder the adsorption of OH^–^ ions and increase the energy barrier for Ni(OH)_2_ oxidation. Consequently, the peak potential of Ni(OH)_2_ oxidation moved slightly to be more positive as the cyclohexanol concentration increased. Figure 5g shows the effect of flow rates of the electrolyte. Results show that increasing the flow rate from 0.6 mL/min to 4.4 mL/min can lead to a slight increase in current densities, with a maximum increase of only 11%. This increase can be attributed to two reasons. One was the decrease in the thickness of the subinterface layer, thereby leading to a faster diffusion of cyclohexanol. Another reason was the faster removal of cyclohexanone from the electrode surface, providing more active redox sites to facilitate the reaction. The investigations above demonstrated that compared to the charge transfer reaction of Ni(OH)_2_/NiOOH and the mass transfer process, the chemical reaction between adsorbed cyclohexanol and nickel oxyhydroxide played a dominant role in the entire oxidation process, acting as the rate-determining step.

### 3.4 Screening the Effects of Surfactants on Cyclohexanol Oxidation

**Figure 6:**
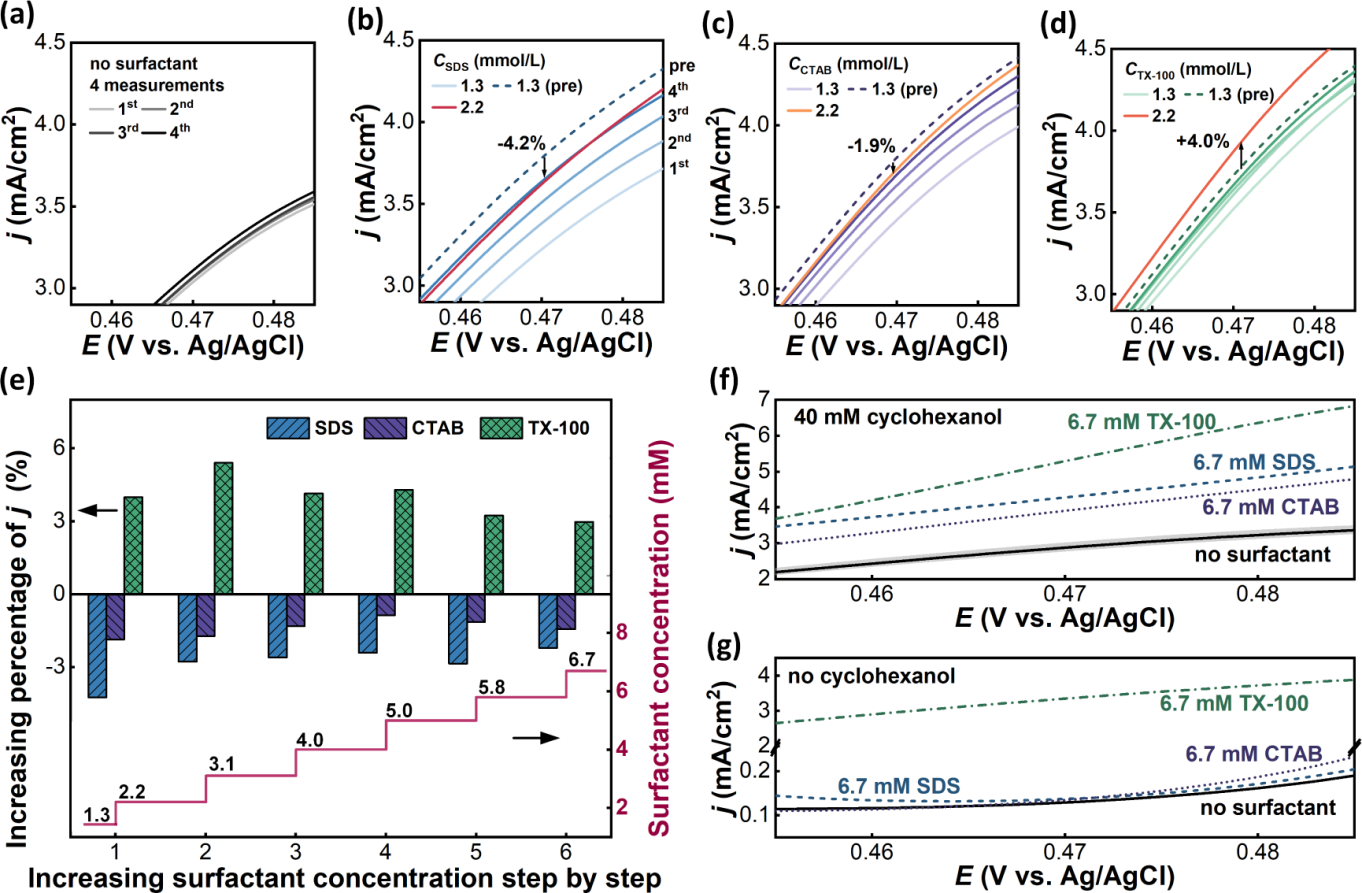
The influences of surfactants on the electrocatalytic oxidation of cyclohexanol on the nickel electrode. (a) Four consecutive LSV measurements at the same experimental conditions using the electrolyte containing 40 mM cyclohexanol without surfactant. (b–d) The effects of increasing concentrations of (b) SDS, (c) CTAB, and (d) Triton X-100 on current densities. The dashed lines represent the predicted fifth LSV curve if the surfactant concentration does not increase, used as a benchmark to evaluate the effect of increasing surfactant concentration. (e) The increasing percentages of current densities when ramping up surfactant concentrations from 1.3 to 6.7 mmol/L step by step, with excluding the interference induced by running measurements. (f) A comparison of the effects of three surfactants on current densities. The black solid curve is the average result from three blank experiments, and the gray area represents the standard error. (g) A comparison of the effects of three surfactants on current densities without cyclohexanol. All experiments were conducted at a scan rate of 2 mV/s and a flow rate of 0.9 mL/min.

Surfactants have been widely used as additives in electrolytes to modify the microenvironment of the electrode–electrolyte interface and modulate electrochemical reaction processes.^34−36^ Suitable surfactants can enhance activity by promoting the adsorption of reactants and increase selectivity by inhibiting side reactions,^37−40^ while unsuitable surfactants may have no or even negative effects.^41,42^ Here, we demonstrated the platform’s ability and advantages for screening the effects of surfactant additives on cyclohexanol electrooxidation. Three surfactants were carefully examined, including the cationic surfactant CTAB, the anionic surfactant SDS, and the nonionic surfactant Triton X-100. A fresh nickel electrode was used for measurements with each surfactant to avoid cross-contamination. CV results indicate that all three surfactants increased the oxidation current density, with Triton X-100 having the most significant effect, as shown in Figure S4. Nonetheless, the CV results for different surfactant concentrations showed poor comparability, probably due to the fluctuations caused by the nonequilibrated adsorption and desorption of surfactants on the electrode surface. To approach a steady-state process, we conducted LSV measurements at a lower scan rate to investigate the effects of surfactant types and concentration variations. It was found that, compared to experiments without surfactants, experiments with surfactants showed increases in current densities between two consecutive LSV measurements, as shown in Figure 6a–d. This suggested that when evaluating the effect of surfactant concentration on current density using LSVs, the interference induced by the measurements should be considered. To address this, five consecutive LSV measurements were conducted for each increase in concentration. The first four LSVs were used to predict the potential fifth curve with surfactant concentration unchanged, by employing a linear regression. The fifth curve was measured after increasing surfactant concentration. The predicted fifth curve, shown by a blue dashed line in Figure 6b, was used as a benchmark to be compared with the actual fifth curve, shown by a red solid line, to evaluate the immediate effect of increasing surfactant concentration.

Results show that conducting LSV measurements resulted in noticeable increases in the current density for experiments with ionic surfactants. This is because, for each LSV, continuously applying potential promoted ionic surfactants to accumulate on the electrode surface and displace water from the surface,^40^ which benefited cyclohexanol adsorption and increased the current density. It was interesting to find that after excluding this interference induced by LSV measurements, increasing the surfactant concentration from 1.3 to 2.2 mmol/L resulted in an immediate decrease in current densities by 4.2% for SDS and 1.9% for CTAB, as indicated by the black arrows in Figure 6b,c. However, for experiments with the nonionic surfactant Triton X-100, increasing concentration led to a 4.0% increase in current density, as shown in Figure 6d. As the concentration increased from 2.2 to 6.7 mmol/L step by step, consistent trends were observed, as shown in Figure 6e, with corresponding LSV profiles shown in Figure S5. Although all three surfactants exhibited a promotional effect on the current densities upon reaching a concentration of 6.7 mmol/L, as shown in Figure 6f, the underlying mechanisms appeared to be different. Basically, ionic surfactants have stronger interactions with the electrode surface, including electrostatic attraction and ionic bonding, while nonionic surfactants interact with the electrode surface typically through relatively weaker forces, such as van der Waals interactions. Thus, ionic surfactants generally exhibit stronger adsorption on electrode surfaces compared to nonionic surfactants.^35^ Therefore, the immediate decrease in current densities could be attributed to an immediate reduction in the surface coverage of cyclohexanol due to the strong competitive adsorption of ionic surfactants. To further clarify the differences in LSV profiles between ionic and nonionic surfactant experiments, LSVs were performed without cyclohexanol, as shown in Figure 6g. Results show that SDS and CTAB had little effect on the Ni(OH)_2_/NiOOH redox step, suggesting that the positive effect of ionic surfactants on current density in cyclohexanol experiments primarily lay in promoting the cyclohexanol oxidation step by favoring the adsorption of cyclohexanol. On the contrary, the addition of Triton X-100 significantly increases the current densities, acting as a reactant like cyclohexanol. This indicated that the increases in current densities in cyclohexanol experiments with Triton X-100 could be attributed to the electrochemical reaction of Triton X-100, rather than the promotion of cyclohexanol oxidation. The experiments here demonstrated the ability of the platform to investigate the surfactant effects on the electrooxidation of cyclohexanol. In addition, owing to the automated microfluidics we developed, LSV measurements and inline surfactant concentration ramping can be performed efficiently, with a reduction of the labor time by 44%, as detailed in Figure S3b. Besides the labor time, the duration of LSV measurements also constituted a significant portion of the total experimental time. This time consumption was determined by the selected LSV method, specifically the scan rate and potential range, and can be significantly reduced severalfold through multiplexing,^43^ an inherent advantage of microfluidics.

## 4 Conclusion

In this work, we developed an automated microfluidics platform for characterizing electrochemical reactions efficiently under well-controlled conditions and applied it to study the electrocatalytic oxidation of cyclohexanol, a power-to-chemical route that could potentially be used for sustainable chemical production. The platform featured microchannel networks integrated with multiple analytical instruments, including pumps, an electrochemical workstation, and a digital microscope, to perform lab functions including electrolyte preparation, reaction control, and characterization, all streamlined through automation. Using the platform, automated electrochemical and optical measurements of cyclohexanol oxidation on Ni electrodes were conducted under different electrolyte compositions and flow rates. We validated that the electrochemical process follows Fleischmann’s mechanism and identified the oxidation of cyclohexanol by nickel oxyhydroxide as the rate-determining step. The effects of surfactant additives were screened by ramping up the surfactant concentrations inline, which revealed that both ionic and nonionic surfactants had a positive effect on the oxidation current density but through different mechanisms. These results demonstrate the significant capability of the developed platform for conducting accurate and rapid electrochemical characterizations. It is readily applied to measure other power-to-chemical processes and serves as a powerful tool for advancing the understanding and progress of sustainable electrosynthesis.

## Supporting Information Available

Supplementary experimental methods, an extended discussion on time savings, additional voltammogram data, and a sample code for demonstration (PDF)
