# Supplementary material for: Automated Microfluidics for Efficient Characterization of Cyclohexanol Electrooxidation for Sustainable Chemical Production
Source: JACS Au. 2025 Mar 4;5(3):1340–9. doi: 10.1021/jacsau.4c01207 (PMC11937964; doi:10.1021/jacsau.4c01207)
Supplement: Supplementary file 1 — au4c01207_si_001.pdf [file au4c01207_si_001.pdf]

Supplementary information

Automated microfluidics for efficient characterization of cyclohexanol electrooxidation for sustainable chemical production

Xiao Liang[1], Mengzheng Ouyang[2], Nigel P. Brandon[2], Jin Xuan[3], Huizhi Wang[1*]

[1]*Department of Mechanical Engineering, Imperial College London, London SW7 2AZ, United Kingdom*

[2]*Department of Earth Science and Engineering, Imperial College London, London, SW7 2AZ, United Kingdom*

[3]*School of Chemistry and Chemical Engineering, Faculty of Engineering and Physical Sciences, University of Surrey, Guildford GU2 7XH, United Kingdom*

*Email: huizhi.wang@imperial.ac.uk

1. Microfluidic mixer

A 3D-printed microfluidic mixer was designed to prepare anolyte and catholyte with various concentrations. Each electrolyte was prepared by mixing two solutions, one was the concentrated solution, and another one was used for dilution. The mixing channel had a compact design of a parallel-flow configuration followed by a serpentine microchannel, as shown in Figure S1(a). Helical microstructures were employed in the serpentine microchannel to enhance the convective mixing, which exhibited an excellent mixing performance compared with the conventional one, as shown in Figure S1(b).

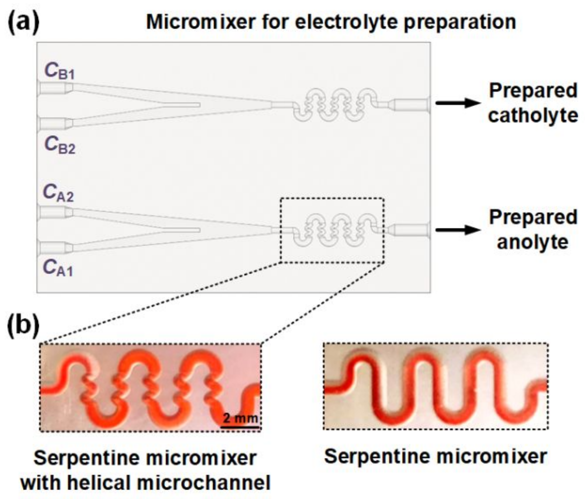

Figure S1. A 3D-printed microfluidic mixer. (a) Schematic of channels in microfluidic mixer. (b) A comparison of the mixing performance in a serpentine micromixer with and without the helical microstructure.

2. The calibrations of flow rate and the dilution factor

The adjustments of flow rates and concentrations were achieved by changing the outlet pressure of four pumps, named as Pump A1, A2, B1 and B2, as shown in Figure 2S(a). Four outlet pressure were set to be P_1, P_2, P_1, P_2, respectively, so the total outlet pressure of Pump A1 and Pump A2 was equal to the total pressure of Pump B1 and Pump B2 in the study. Thus, the flow rate of anolyte Q_{WE} and the flow rate of catholyte Q_{CE} were equal and can be calculated by,

$$Q_{\mathrm{WE}} = Q_{\mathrm{CE}} = \frac{1}{2} Q_{\mathrm{T}} = \frac{1}{2}\left(\frac{P_{\mathrm{A1}} + P_{\mathrm{A2}} + P_{\mathrm{B1}} + P_{\mathrm{B2}} - P_{\mathrm{o}}}{R_{\mathrm{T}}}\right) \quad \text{(S1)}$$

where R_{T} is flow resistance of the entire flow path; Q_{T} is the flow rate at the outlet.

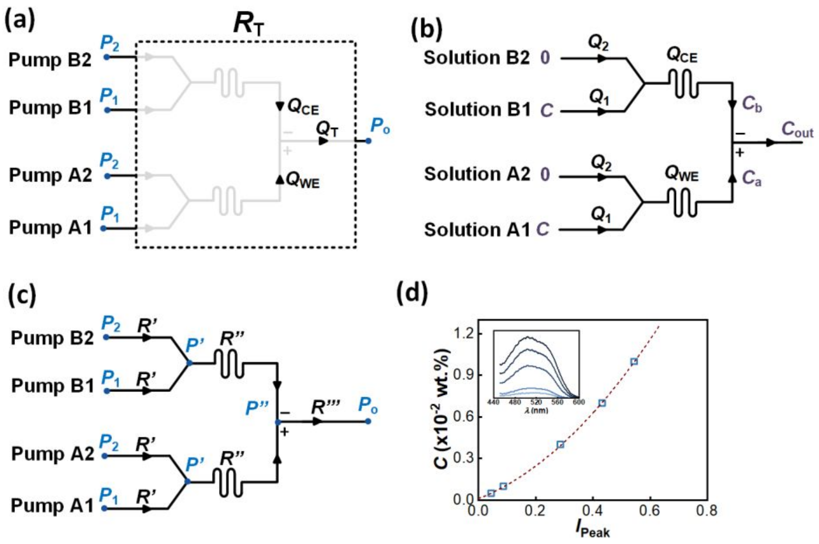

Figure S2. The calibrations of flow rate and dilution factor. (a)-(c) Schematic of the flow path design with key parameters, including pressure, flow rates, and concentrations. (d) A calibration curve of Allura red dye measured by a near-infrared and visible spectroscopy. The inset graph shows the spectra with different dye concentrations.

To examine the relationship between the prepared electrolyte concentration and outlet pressure, Allura red dye was added in two solutions A1 and B1 with the concentrations of C to simulate the electrolyte component of interest, and two deionized water solutions A2 and B2 were used to simulate the dilution solutions, as shown in Figure S2(b). The concentrations of diluted anolyte and catholyte were C_a and C_b. The concentration of solution at the outlet was C_{out}. According to the principal of mass conservation and Poiseuille's Law,

$$C_{out} = C_a = C_b = \frac{Q_1}{Q_1 + Q_2} C \tag{S2}$$

$$Q_1 = \frac{P_1 - P'}{R'} \tag{S3}$$

$$Q_2 = \frac{P_2 - P'}{R'} \tag{S4}$$

$$Q_{\mathrm{WE}} = Q_{\mathrm{CE}} = Q_1 + Q_2 = \frac{P' - P''}{R''} = \frac{1}{2}\frac{P'' - P_{\mathrm{o}}}{R'''} \tag{S5}$$

where Q_1 and Q_2 are the flow rates in the four initial branch paths; R', R'' and R''' are the flow resistances of the branch flow paths; P' and P'' are the pressure at the joint points of the branch paths, as shown in Figure S2(c). After derivation, the concentration of the prepared anolyte C_{a} can be calculated by,

$$C_{\mathrm{a}} = \rho C = \left[\frac{(R' + 2R'' + 4R''')}{R'(P_1 + P_2) - 2R'P_{\mathrm{o}}} P_1 - \frac{(R'' + 2R''')(P_1 + P_2) + R'P_{\mathrm{o}}}{R'(P_1 + P_2) - 2R'P_{\mathrm{o}}}\right] C \tag{S6}$$

where ρ is the dilution factor. This equation suggests that ρ has a linear relationship with the outlet pressure P_1 with P_1+P_2 constant. Thus, ρ can be adjusted by changing P_1 with P_1+P_2 unchanged. Different values of P_1+P_2 can lead to different linear relationships between ρ and P_1, as shown in Figure 3(d). The dye concentration in the prepared solution was measured by a near-infrared and visible spectroscopy (Model 4250, Hinalea VNIR camera) and the calibration curve is shown in Figure S2(d).

3. Time savings with using the automated microfluidic platform

The labor time consumption of the automated and manual experiments was compared in Figure S3. Results show that compared to the manual experiments using conventional H-cell, the automated microfluidic platform significantly reduced the labor time in the electrolyte preparation before the measurements, and the operations during the measurements, such as electrolyte or electrode replacement, device reassembly, and parameter adjustments. Overall, the automated microfluidic experiments saved nearly half of the labor time compared to manual experiments, highlighting its significant advantage in accelerating experiments, making it ideal for efficient electrochemical characterizations. Besides the labor time, the duration of electrochemical measurements also constituted a significant portion of the total experimental time. This time consumption can be significantly reduced by performing parallel electrochemical measurements through multiplexing, an inherent benefit of microfluidics [43].

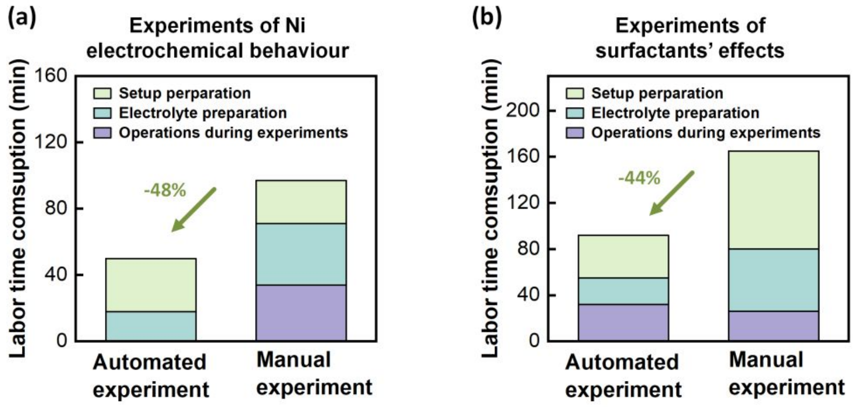

Figure S3. Comparison of the labor time consumption between automated and manual experiments. (a) Labor time consumed in CV experiments, corresponding to Figure 4. (b) Labor time consumed in LSV experiments, corresponding to Figure 6. Labor time in manual experiments were estimated using an H-cell with stirring and a filter paper as the separator.

4. Additional voltammograms with different surfactants

The CV profiles with adding surfactants are shown in Figure S4. The result shows that all three surfactants increase the oxidation current density, with Triton X-100 having the most significant effect.

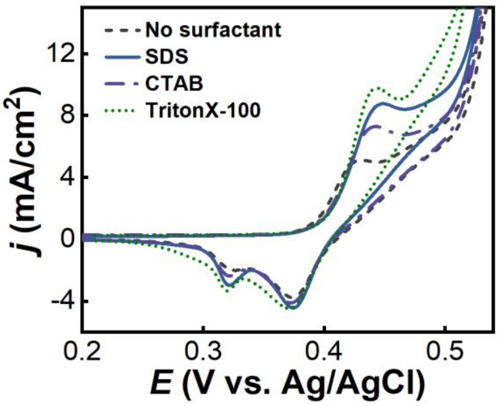

Figure S4. CV profiles with the addition of surfactant additives. All experiments were conducted using anolytes of 0.5 M NaOH solution containing 4 mM surfactant and 40 mM cyclohexanol, with the flow rate of 0.9 mL/min. The scan rate is 10 mV/s.

The LSV profiles with increasing surfactant concentrations step by step are shown in Figure S5. Although all three types of surfactants led to increased current densities in the final results, the underlying mechanisms appeared to be different. The addition of ionic surfactants, especially for SDS, led to an immediate decrease in current densities, but resulted in an increase in current densities after each LSV measurement. The addition of nonionic surfactant Triton X-100 increased the current densities and running LSV measurement had little influence on current densities. These indicated the different mechanisms of ionic and nonionic surfactants affecting the electrode-electrolyte interface during cyclohexanol oxidation.

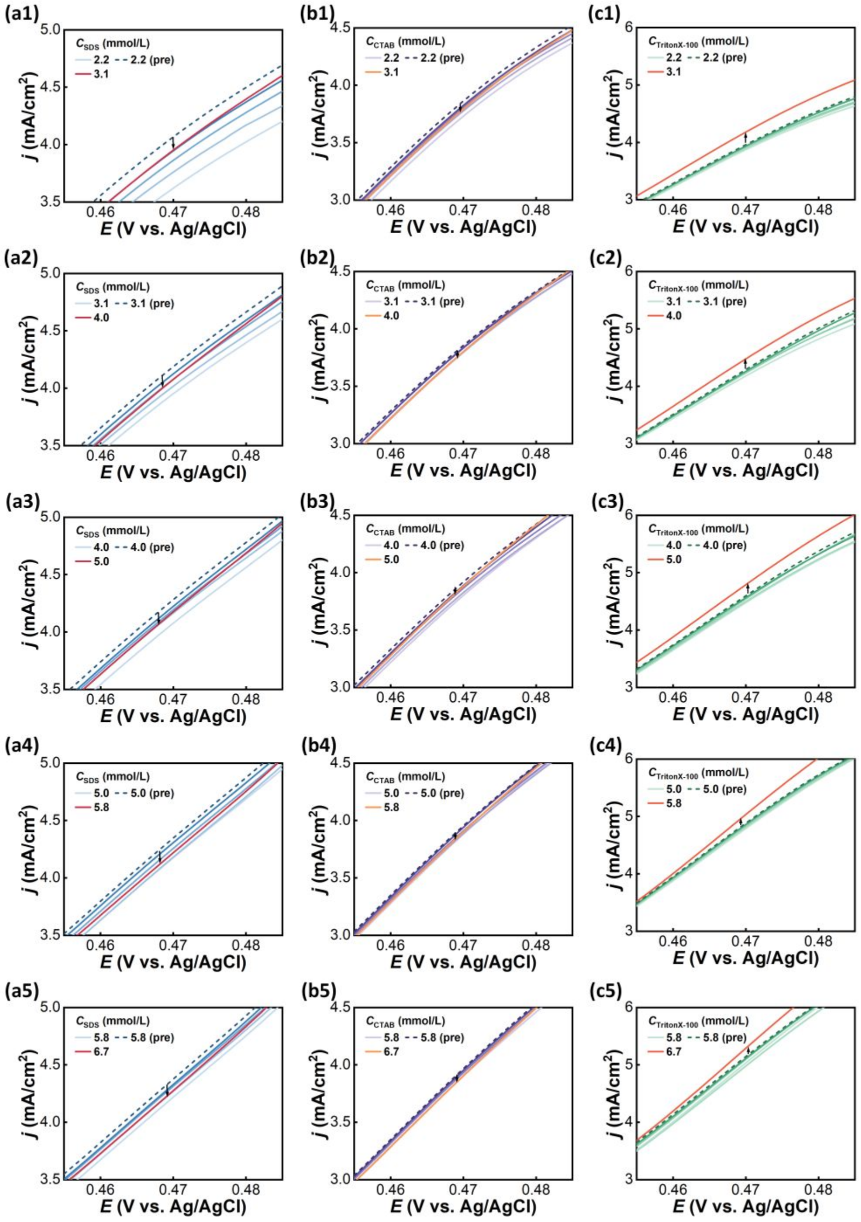

Figure S5. LSV profiles with increasing surfactant concentrations step by step. The surfactant concentrations were increased from 2.2 mmol/L to 6.7 mmol/L for (a) SDS, (b) CTAB, and (c) Triton X-100. NaOH concentration was 0.5 M and cyclohexanol concentration was 40 mM. All experiments were conducted at the scan rate of 2 mV/s and the flow rate of 0.9 mL/min.

5. Code for platform control

The Python code developed for this experimental platform integrated multiple hardware components to control and automate the process. The key functionalities included pump control, electrochemical workstation operation and camera control, achieved by using the Fluigent Software Development Kit, Hard Potato [44] and OpenCV packages, respectively. The modular design of the code allowed for easy customization and efficient operation, making it adaptable for a wide range of experimental setups. The code for the experiments with adjusting the electrolyte concentration was attached below as a showcase.

```
# Pump package
from __future__ import print_function
import time
from Fluigent.SDK import fgt_init, fgt_close
from Fluigent.SDK import fgt_set_pressure, fgt_get_pressure,
fgt_get_pressureRange
from Fluigent.SDK import fgt_get_pressureChannelCount

# Electrochemical workstation package
import hardpotato as hp
import os

# Camera package
import cv2
import time
import threading


#### Camera setup ####

## Define a function to capture video from the camera
def capture_video(Videosave_path, capture_time, codec="XVID"):
    # Open the default camera
    cap = cv2.VideoCapture(0)

    # Check if camera opened successfully
    if not cap.isOpened():
        print("Error: Could not open camera.")
```

```
        return

    # Create VideoWriter object
    width = cap.get(cv2.CAP_PROP_FRAME_WIDTH)
    height = cap.get(cv2.CAP_PROP_FRAME_HEIGHT)
    fps = cap.get(cv2.CAP_PROP_FPS)
    brightness = cap.get(cv2.CAP_PROP_BRIGHTNESS)
    contrast = cap.get(cv2.CAP_PROP_CONTRAST)
    frame_size = (int(width),int(height))

    cap.set(cv2.CAP_PROP_FRAME_WIDTH, int(width))
    cap.set(cv2.CAP_PROP_FRAME_HEIGHT, int(height))
    cap.set(cv2.CAP_PROP_BRIGHTNESS, brightness)
    cap.set(cv2.CAP_PROP_CONTRAST,contrast)

    fourcc = cv2.VideoWriter_fourcc(*codec)
    out = cv2.VideoWriter(Videosave_path, fourcc, fps,
frame_size)

    start_time = time.time()

    try:
        while True:
            ret, frame = cap.read()
            if not ret:
                print("Error: Could not read frame.")
                break

            # Save the captured frame to the video file
            out.write(frame)

            # Show the frame
            cv2.imshow('frame', frame)

            # Check if 'q' is pressed or the capture duration is
reached
            if cv2.waitKey(1) & 0xFF == ord('q') or (time.time()
- start_time) > capture_time:
                break
    finally:
        # Release everything if job is finished
        cap.release()
        out.release()
        cv2.destroyAllWindows()

# Define a function for the first task
def capture_video_task():
    capture_video(Videosave_path, capture_time)

# Define a function for the second task
def cv_run_task():
    cv.run()

####-------------------------------------####


#### Pressure pumps setup ####
```

```
# Initialize the session
fgt_init()

# Set and read pressure
fgt_set_pressure(0, 0) # mbar is the default unit

# Set differences in pressures for pumps to control dilution
factors
pressure_step_A1 = [-2,-1,0,1,2] # pump A1, mother solution:
anolyte that flows by the working electrode: +- x mbar
pressure_step_A2 = pressure_step_A1[::-1] # pump A2, dilution
solution: anolyte that flows by the working electrode: +- x mbar
pressure_step_B = 0 # pump B1 & B2: catholyte that flows by the
counter electrode: constant concentration

# Set the base pressure
pressure_A = 20 #pressure_A=pressure_A1+pressure_A2
pressure_B = pressure_A #pressure_B=pressure_B1+pressure_B2

# Set pressure for pumps to standby
pressure_standby = 20

# Set times for each measurement (second)
air_removing_time = 10
waiting_time = 30
rest_Time = 10

####-------------------------------------####


#### Electrochemical workstation setup ####

# Select the potentiostat model to use:
model = 'chi760e'
# Path to the chi software, including extension .exe
path = 'C:\Software\Workstation\chi660e\chi660e.exe'

# Folder where to save the data
dir_path = os.path.dirname(os.path.abspath(__file__))
folder='Test'
folder_path = os.path.join(dir_path, folder)
os.makedirs(folder_path, exist_ok=True)

# Initialize electrochemical workstation
hp.potentiostat.Setup(model=model, path=path, folder=folder)

# Set electrochemical workstation parameters
Eini = 0.2     # V, initial potential
Ev1 = 0.55       # V, first vertex potential
Ev2 = 0.2      # V, second vertex potential
Efin = 0.2     # V, final potential
sr = 0.01        # V/s, scan rate
dE = 0.002      # V, potential increment
nSweeps = 1     # number of sweeps, 0.999 ==> LSV, 1 ==> 1 CV
cycle
sens = 1e-4     # A/V, current sensitivity
```

```
####------------------------------------####


#### Main ####

# Set pressure for bubbles removing before experiments
pressure_removing_bubbles = 100

fgt_set_pressure(0, pressure_removing_bubbles)
fgt_set_pressure(1, pressure_removing_bubbles)
fgt_set_pressure(2, pressure_removing_bubbles)
fgt_set_pressure(3, pressure_removing_bubbles)

time.sleep(air_removing_time)

# Begin main experimental loop
for i in range(len(pressure_step_A1)):
    pressure_A1= pressure_A + pressure_step_A1[i] # anolyte that
flows by the working electrode (mother solution)
    pressure_A2= pressure_A - pressure_step_A1[i] # anolyte that
flows by the working electrode (dilute solution)
    pressure_B1= pressure_B # catholyte that flows by the counter
electrode
    pressure_B2= pressure_B # catholyte that flows by the counter
electrode

    # input pressure parameter into pumps
    fgt_set_pressure(0, pressure_A1)
    fgt_set_pressure(1, pressure_A2)
    fgt_set_pressure(2, pressure_B1)
    fgt_set_pressure(3, pressure_B2)

    # wait before running
    time.sleep(waiting_time)

    # set CV measuremnents
    msr = sr*1000
    mEini = Eini*1000
    mEv1 = Ev1*1000
    fileName =
'Pa1_{:.0f}mbar'.format(pressure_A1)+'_Pa2_{:.0f}mbar'.format(pre
ssure_A2) # base file name for data file
    header = 'CV'   # header for data filef"file_{x}.txt"
    print(fileName)

    # initialize electrochemical experiment
    cv = hp.potentiostat.CV(Eini, Ev1,Ev2, Efin, sr, dE, nSweeps,
sens, fileName, header)

    # set camera
    Videosave_path = os.path.join(folder_path, fileName) + '.avi'

    if nSweeps < 1:
        capture_time = (Ev1-Eini)/sr*nSweeps + 2
    elif nSweeps == 1:
        capture_time = (Ev1-Eini)/sr*nSweeps*2 + 2
```

```
    elif nSweeps > 1:
        capture_time = (Ev1-Eini)/sr*nSweeps + 2


    ## Run electrochemical experiments and video recording
simultaneously

    # Create threads for each task
    capture_video_thread =
threading.Thread(target=capture_video_task)
    cv_run_thread = threading.Thread(target=cv_run_task)

    # Start both threads
    capture_video_thread.start()
    cv_run_thread.start()

    # Wait for both threads to finish
    capture_video_thread.join()
    cv_run_thread.join()

    # Wait after running
    time.sleep(rest_Time)

# Set pumps to standby after completing experiments
fgt_set_pressure(0, pressure_standby)
fgt_set_pressure(1, pressure_standby)
fgt_set_pressure(2, pressure_standby)
fgt_set_pressure(3, pressure_standby)
```

Reference

43. Liu, W., and J. M. Lin. Online monitoring of lactate efflux by multi-channel microfluidic chip-mass spectrometry for rapid drug evaluation. *ACS Sens.* **2016**, *1* (4), 344–347.

44. Rodriguez, O.; Pence, M. A.; Rodriguez-Lopez, J., Hard Potato: a python library to control commercial potentiostats and to automate electrochemical experiments. *Anal Chem* **2023**, 95 (11), 4840-4845.
